# Natural Magnesium-Enriched Deep-Sea Water Improves Insulin Resistance and the Lipid Profile of Prediabetic Adults: A Randomized, Double-Blinded Crossover Trial

**DOI:** 10.3390/nu12020515

**Published:** 2020-02-18

**Authors:** Ji Yeon Ham, Yun Hee Shon

**Affiliations:** 1 Department of Laboratory Medicine, Kyungpook National University Hospital, 130 Dongdukro Jung-gu, Daegu 41944, Korea; twinkle-hj@hotmail.com; 2 Bio-Medical Research Institute, Kyungpook National University Hospital, 135 Dongdukro Jung-gu, Daegu 41940, Korea

**Keywords:** balanced deep-sea water, clinical trial, diabetes mellitus, glucose metabolism, lipid profile, magnesium, prediabetes

## Abstract

Previous in vitro and in vivo studies have shown that the antidiabetic effect of balanced deep-sea water (BDSW) works through the suppression of hyperglycemia and improvement of glucose tolerance. Based on these promising results, we conducted an eight week randomized, double-blinded crossover trial of the effects of BDSW in prediabetic adults. The subjects consumed 440 mL of BDSW (hardness 4000) per day, and maintained an otherwise normal lifestyle and diet throughout. Efficacy assessments were made by measuring fasting glucose, postprandial glucose, fasting insulin, homeostasis model assessment for insulin resistance (HOMA-IR), C-peptide, glycosylated hemoglobin, lipid metabolism indicators, and physical metrics, along with safety assessments. Fasting insulin and HOMA-IR values of the BDSW group were significantly lower than those of the placebo group after eight weeks of BDSW ingestion. Total cholesterol and low-density lipoprotein–cholesterol were also significantly decreased in the BDSW group after eight weeks of BDSW ingestion compared with the placebo group. There were no statistically and clinically meaningful changes in adverse events, physical examination, laboratory medicine examination, or vital signs of the BDSW intake group. These results suggested that the intake of BDSW in prediabetic adults can improve glucose metabolism and lipid profiles and is safe for human consumption.

## 1. Introduction

Diabetes mellitus is the most common endocrine disorder in modern people. According to data released by the International Diabetes Federation in 2017, about 500 million people worldwide suffer from diabetes. Thus, diabetes is not just a health crisis, but a global disaster [1]. Diabetes itself is dangerous and unless adequate treatment is provided, diverse and serious complications such as diabetic retinopathy [2], stroke [3], chronic renal failure [4], angina pectoris [5], and myocardial infarction [6] can cause a decrease in quality of life and lead to patient death.

Deep-sea water is low-temperature water that exists at a depth of more than 200 m. It is not exposed to sunlight, so the water contains almost no biodegradable organic components. Deep-sea water contains a large number of natural minerals such as Mg^2+^, Ca^2+^, Na^+^, Zn^2+^, and K^+^; thus it represents a rich marine resource that is clean and contains natural minerals [7].

Magnesium (Mg) is the fourth abundant mineral in the human body and is an important metabolic electrolyte. It is known as a cofactor in more than 300 enzyme systems that regulate a variety of body biochemical reactions, including energy generation [8], enzyme activation [9], cardiovascular system [10], membrane function [11], and nutritional metabolism [12]. As magnesium has many functions in the human body, it also plays an important role in the prevention and treatment of various diseases. Studies have shown that hypomagnesemia results in patients having a high risk of multiple metabolic and cardiovascular diseases [13,14,15]. The effectiveness of magnesium supplements to improve glycemic and insulin-sensitivity parameters in pre-diabetic and diabetic participants has already been proposed in previous clinical trials [12,16,17]. Therefore, ingestion of deep-sea water containing large amounts of natural magnesium may be helpful to improve symptoms in diabetic patients. Previous reports have shown that the physiological activities of deep-sea water included improvements to arteriosclerosis [18], improperly raised blood pressure [19], and atopic dermatitis [20] in animal studies. However, there has not yet been any systematic study on the effects of deep-sea water on diabetic symptoms or its mechanism of action in the human body.

Previously, our studies on the antidiabetic effects of balanced deep-sea water (BDSW) have shown that hyperglycemia and glucose tolerance were improved by accelerating glucose uptake, along with the suppression of glucose production-related genes, and a recovery of pancreatic function in experimental animal models of obesity and non-obesity type diabetes mellitus [21,22]. Based on the efficacy of BDSW in cell and animal experiments, the clinical trial presented here was conducted to evaluate the efficacy of BDSW on glycemic and insulin-sensitivity parameters, as well as its safety.

## 2. Materials and Methods

### 2.1. Ethics

The clinical trial was carried out in accordance with the Declaration of Helsinki at the Clinical Trial Center for Functional Foods, Chonbuk National University Hospital in South Korea. It was reviewed by the Institutional Review Board (IRB) of Chonbuk National University Hospital and its implementation was approved (IRB No. CTCF2_2016_DSW, date. 2016.04.28). The trial was performed in compliance with Good Clinical Practice. The progress of the trial was monitored at the hospital and was carried out by Bio Food Story (Contract Research Organization; Ogol 4–gil 16, Dukjin-gu, Jeonju, Jeollabuk-do).

After being fully informed of the purpose of the study, as well as any possible adverse reactions, all participants gave their informed consent in signed document format before they participated in the study.

### 2.2. Subject

All subjects who participated in the clinical trial met the following criteria: adults between 19 and 70 years of age, with no history of diabetes mellitus within the last 3 months, 75 g oral glucose tolerance test (OGTT) and fasting blood glucose levels of 100–125 mg/dL, and 2 h postprandial blood glucose levels of 140–199 mg/dL. All study participants heard and understood the full details of this study, voluntarily decided to participate, and agreed in writing to comply with the precautions. The criterion for exclusion of study subjects is available as Supplementary Materials (Supplementary File 1).

### 2.3. Preparation of Product (BDSW)

Deep-sea water was collected at a depth of 0.5 km and a distance of 6.7 km off Oho-Ri, Goseong (Gangwon-Do, Korea). It was then filtered through a microfiltration membrane (Synopex, Pohang, Korea), and deep-sea water mineral extracts and desalinated water were obtained using a reverse osmosis system (Vontron Technology Co., Ltd., Beijing, China). BDSW was used in this trial by mixing desalinated water with magnesium and calcium at a constant ratio (magnesium: calcium = 3:1), producing a hardness of 4000 as shown in (Table 1) [23]. The mineral content of deep-sea water is defined as hardness and calculated internationally according to the following formula: hardness = Mg (mg/L) × 4.1 + Ca (mg/L) × 2.5.

In this study, the functional ingredient of the product was set as magnesium (Mg). According to the Korean daily nutritional intake standard, the recommended daily intake of magnesium for adults is 350 mg. The magnesium content of commercially available mineral products is 400–500 mg/day; thus, the amount proposed in this study (350 mg) is within that range. Therefore, the intake suggested for this study was judged as likely to present no safety problems. When the amount of magnesium was set at 350 mg, based on a water hardness of 4000 in BDSW, the volume of the product consumed by each subject per day was 440 mL (Table 1). The placebo was made with purified water as the main ingredient and was approximately equal in taste, weight, and calories to BDSW (Table 1).

### 2.4. Method of Product Intake

The subjects were then randomly assigned to group A or group B on the first day of the first visit. The first trial product was taken for eight weeks according to the order of intake of the trial product by the group, and then the second trial product was taken for eight weeks after a four-week break (Table 2).

### 2.5. Study Design

**Figure A1:**
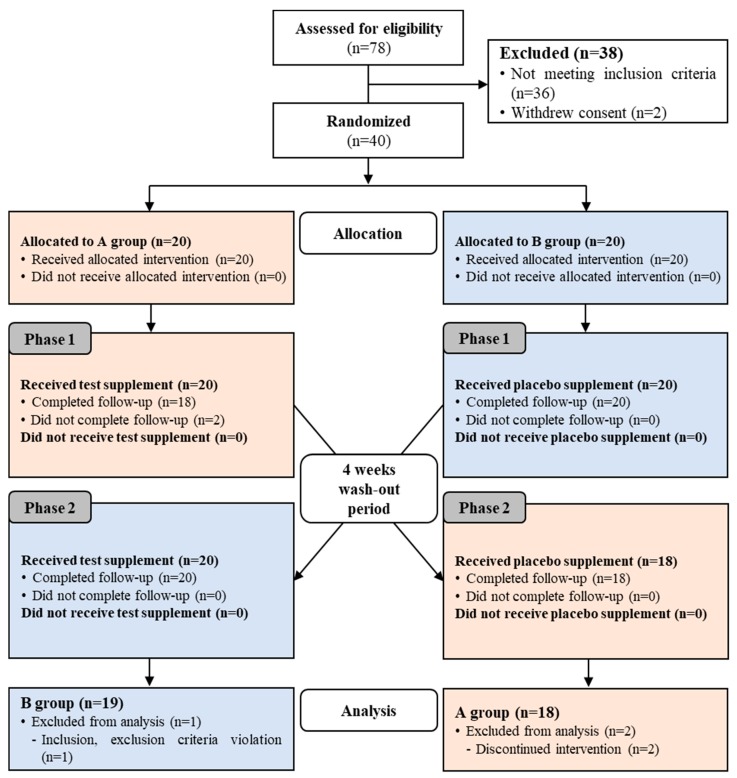
CONSORT 2010 flow diagram.

This study was an eight-week, randomized, double-blind, placebo-contrast, crossover study to assess the efficacy and safety of BDSW in improving glucose metabolism. This clinical trial was registered at ClinicalTrials.gov with trial registration number NCT03956914. The CONSORT flow diagram (Figure A1) and checklist (Table A1) are provided in the Appendix A.

Randomization was used in the double-blind phase to avoid bias when assigning subjects and to increase the effectiveness of statistical comparisons between treatment groups by balancing the known or unknown characteristics of the subjects (demographic and baseline characteristics) for each treatment group. Double-blind administration was used to reduce potential bias in data collection and evaluation at the end of the clinical trial. In order to maintain the double-blind procedure during the clinical trial period, neither the researcher nor the subject should be able to know the subject’s randomization information. Therefore, the randomization code was only in the possession of Kyungpook National University Hospital. In addition, no subject was released from double-blinding until the trial was completed, and data were locked. If double-blinding was lifted, the date, time, and reason for the release were documented in the case record and in the appropriate documentation. A copy of the blinding code release confirmation document received from Kyungpook National University Hospital was kept in the supporting documents, which are available upon request.

A summary of the clinical trial schedule is provided in the Supplementary Materials (Supplementary File 2). The efficacy evaluation metrics were blood glucose-related index, blood lipid index, and anthropometric index. After fasting for 12 h, blood glucose levels were measured by taking blood from the upper arm vein after ingesting the clinical trial product and a 75 g glucose solution. At the screening visit, blood samples were collected before the ingestion of a 75 g glucose solution (0 min) and 120 min after the ingestion of a 75 g glucose solution without consumption of the clinical trial product. At the first, second, third, and fourth visits, blood samples were taken at 0, 30, 60, 90, and 120 min after ingesting trial products and 75 g glucose solutions. The clinical trial product was taken just before the consumption of 75 g of glucose.

Blood for the measurement of fat indicators was collected from veins such as the superior vena cava while maintaining 12 h fasting; total cholesterol (TC), triglycerides (TG), low-density lipoprotein–cholesterol (LDL-C), and high-density lipoprotein–cholesterol (HDL-C) were measured.

The anthropometric indicators included the measurement of height, weight, body fat, body fat percentage, body mass index, waist circumference, hip circumference, and waist-to-hip ratio.

The safety evaluation items are adverse reactions, physical examination, laboratory medicinal examination, and vital signs. Adverse events were recorded in detail on the timing, duration, severity, outcome, and causation of adverse events. Physical examinations included interviews, inspection, testing using a stethoscope, percussion, and palpation. Laboratory medicinal examinations were carried out with 12 h fasting. Blood was drawn from the veins such as the upper arm vein, and the following items were examined. A general blood trial was used to measure white blood cells (WBC), red blood cells, hemoglobin, hematocrit, and platelets. Blood chemistry trials included alkaline phosphatase, gamma-glutamyl transferase, aspartate aminotransferase, alanine aminotransferase, total bilirubin, total protein, albumin, blood urea nitrogen, creatine, and estimated glomerular filtration rate (eGFR) measurements. A urine trial was used to measure the specific gravity, pH, WBC, nitrite, protein, glucose, ketone, urobilinogen, bilirubin, and occult blood. Vital signs were measured to calculate blood pressure and pulse rate.

### 2.6. Statistical Analysis

Statistical analysis system 9.4 for Windows was used for analysis, and the statistical significance level for all measures was set at 5% or less. The homogeneity trial of the baseline value of the efficacy and safety evaluation items between intake groups was carried out using an independent t-test. The difference between intake groups and within intake groups was analyzed by applying an RM-ANOVA or linear mixed effect model for repeated measurement of the validity and safety assessment metrics. Adverse events that occurred during the trial period were tested using a chi-square or Fisher’s exact test.

## 3. Results

### 3.1. Subjects

A total of 40 subjects were enrolled, with 20 people per allotted group. Since the study followed a cross-design, the total number of people to be analyzed was 80. Among the registered subjects, there were two dropouts, one registrant who violated the selection criteria, and 38 subjects who completed the test (Figure A1). The main analytical group for validation was performed in 37 subjects who completed the study, followed the clinical protocol, and had at least one measure of the main endpoint variable (Figure A1).

Demographic data were analyzed for 40 subjects in the study group who consumed the test product at least once during the trial (Supplementary File 3). As a result of analyzing the baseline of the efficacy evaluation items, there was no statistically significant difference between the two groups in all items (Table 3).

### 3.2. Effects on Glycemic and Insulin-Sensitivity Parameters

Fasting plasma glucose (FPG) and postprandial plasma glucose (PPG) were measured at baseline and eight weeks after the ingestion of test products in the first (0–8 weeks) and second (12–20 weeks) periods. During each clinic visit, blood glucose was measured at baseline and after (30, 60, 90, and 120 min) ingestion of a 75 g glucose solution using the OGTT. The blood glucose response area was shown using a plasma glucose concentration-time curve area, which increased compared to baseline from the start of intake to the point of collection for a two hour period.

The results of blood glucose analysis were summarized in Table 4. There were no statistically significant differences in FPG, PPG, and glucose area under the curve (iAUC) between the two groups (*p* > 0.05).

Fasting plasma insulin (FPI), the homeostasis model assessment for insulin resistance (HOMA-IR), C-peptide, and type A1c glycosylated hemoglobin (HbA1c) were all measured at baseline and eight weeks after ingestion of test products in the first (0–8 weeks) and second (12–20 weeks) periods. The fasting insulin (*p* = 0.042) and HOMA-IR (*p* = 0.049) values of the BDSW group were significantly lower than those of the placebo group after eight weeks of ingestion. C-peptide and HbA1c showed no statistically significant differences between the two groups (*p* > 0.05) (Table 5).

### 3.3. Effect on Lipid Profiles

Serum lipid profiles, TC, TG, LDL-C, and HDL-C were measured at baseline and eight weeks after the ingestion of test products in the first (0–8 weeks) and second (12–20 weeks) periods. As a result, TC (*p* = 0.006) and LDL-C (*p* = 0.003) in the BDSW group were significantly decreased after eight weeks of ingestion of BDSW compared with the placebo group (Table 6).

### 3.4. Dietary Intake Analysis

In order to evaluate the food and nutrient intakes of the subjects during clinical trial participation, a dietary intake survey was conducted at baseline and after eight weeks of ingestion of the test product. The dietary data sheets were prepared according to the meal recording method and were analyzed using CAN Pro 4.0®. As a result, there was no statistically significant difference between the two groups (*p* > 0.05) (Table 7).

### 3.5. Safety Evaluation

A thorough safety evaluation was conducted in the 40 subjects who participated in the clinical trial and consumed the test product at least once (safety group). Table 8 summarizes the results of the laboratory medicinal examinations (hematology, blood biochemistry, and urinalysis) measured before and eight weeks after the ingestion of test products in the first (0–8 weeks) and second (12–20 weeks) periods. There was no statistically significant difference between the two groups (*p* > 0.05). There were no clinically meaningful anomalies or changes according to the ingestion of the products for clinical trials.

Vital signs (systolic blood pressure, diastolic blood pressure, and pulse rate) were measured at baseline and eight weeks after the ingestion of test products in the first (0–8 weeks) and second (12–20 weeks) periods. There were no statistically significant differences between the two groups (*p* > 0.05) and no clinically meaningful changes according to the consumption of clinical trial products (Table 9).

## 4. Discussion

The purpose of this study was to investigate the effects of the regular daily intake of magnesium-rich BDSW on improving glucose and insulin-sensitivity parameters, as well as the overall lipid profile in prediabetes subjects. We found that fasting insulin, HOMA-IR, TC, and LDL-C levels of the magnesium-rich BDSW group were significantly lower than those of the placebo group after eight weeks of ingestion. However, there was no statistically significant difference in fasting glucose, postprandial glucose, iAUC, C-peptide, or HbA1c between the two groups. The subjects of this study were not restricted in their dietary preferences. Such variation in the test subjects may have resulted in our inability to detect clear statistically significant differences in fasting glucose, postprandial glucose, or HbA1c, mainly due to individual differences in the investigated blood glucose parameters.

Magnesium is required for both the proper utilization of glucose and insulin signaling [17]. Cellular magnesium plays a key role in the insulin-mediated glucose uptake. Its deficiency contributes to insulin resistance and a decreased utilization of cellular glucose [17]. It has been reported that hypomagnesemia can lead to a defective activity of tyrosine kinase and can modify insulin sensitivity by influencing the activity of the insulin receptor after binding or by influencing the intracellular signaling and processing [24]. There is an additional hypothesis that magnesium deficiency has deleterious effects on glucose metabolism due to an impairment both in insulin secretion and its action, contributing to the development of type 2 diabetes (T2D) [25,26,27].

Many studies have shown that magnesium supplements improve metabolic metrics related to insulin sensitivity [28,29,30,31]. In a study of Chinese populations, serum magnesium levels were lower in subjects with fasting glucose disorders, impaired glucose tolerance, or T2D than in healthy controls [32]. Zhao-min Liu and Suzanne C. Ho showed that serum magnesium levels were associated with prediabetes in postmenopausal women [33]. Previous observational studies have shown that those who take more magnesium without pre-existing diabetes have a lower risk of developing diabetes [34]. It has also been found that ingesting a magnesium-rich diet helps prevent the risk of developing diabetes in prediabetic patients [17,35]. In a meta-analysis of double-blind randomized controlled trials on the subject of prediabetes, it was found that oral magnesium supplementation reduces HOMA-IR [36]. In a search of several electronic databases, there were no reports of reduced plasma glucose as a primary endpoint in randomized placebo-controlled clinical trials using magnesium supplementation in prediabetic patients [36]. Consistent with such reports, subjects in this study who consumed magnesium-rich BDSW supplements had significantly reduced HOMA-IR indices and no changes to glucose levels.

With reference to available data on this subject, the proportion of prediabetic patients increases in groups with hypomagnesemia and/or low levels of magnesium intake [37,38,39,40,41,42]. These studies support the hypothesis that magnesium deficiency is related to the occurrence of glucose disease. Our findings, and most findings from other studies on related topics [28,29,30,31], demonstrate the efficacy of magnesium supplements in improving blood glucose conditions in prediabetes patients. Such evidence without a mechanism of action clearly warrants further study.

According to recommendations provided by the International Diabetes Federation [1], once diabetes is diagnosed, care must be taken to reduce the risk of future disease. In that sense, promoting a healthy lifestyle is essential. The inclusion of magnesium-rich BDSW supplements in the pharmacological treatment of diabetes can now be predicted to be beneficial, so actively seeking a healthy lifestyle and maintaining an appropriate serum magnesium level using magnesium-rich BDSW intake is a logical course of action. Our results support the hypothesis that oral magnesium-rich BDSW supplementation improves insulin-sensitivity metrics in prediabetes and could be used to establish a public health strategy to reduce the incidence of diabetes.

Dyslipidemia in patients with diabetes plays an important role in promoting atherosclerosis and is known to contribute to the risk of cardiovascular disease. Considering the prevalence and risk of diabetes-related cardiovascular disease, it is necessary to evaluate the lipid profile of prediabetic patients. Therefore, we also studied the impacts of magnesium-rich BDSW on cardiovascular health parameters that may be secondary to glucose metabolism impairment. Our findings showed amelioration in the lipid profiles of people who take magnesium-rich BDSW supplements, and are consistent with previous studies showing that magnesium supplements have a positive impact on lipid response [31,43,44].

In conclusion, our current results demonstrated the efficacy of magnesium-rich BDSW supplements in improving insulin-sensitivity parameters and lipid profiles in patients with prediabetes. We also established that BDSW is completely safe for human consumption.

## Figures and Tables

**Table:** 

	Component	BDSW		Placebo	
		Mixing Ratio (%)	Usage (g)	Mixing Ratio (%)	Usage (g)
Main component	BDSW (hardness 4000)	97.9	430.8	-	-
	Purified water	-	-	96.8	425.9
Minor component	Dextrin	-	-	1.1	4.8
	Cranberry (concentrated solution)	2.0	8.8	2.0	8.8
	Cranberry scent	0.1	0.4	0.1	0.4
Total		100	440	100	440

BDSW, balanced deep-sea water.

**Table:** 

Mineral	BDSW (mg/L)
Magnesium (Mg)	813
Calcium (Ca)	275
Potassium (K)	20
Sodium (Na)	41
Overall hardness ^1)^	4000

BDSW, balanced deep-sea water. ^1)^ 813 (mg/L) × 4.1 + 275 (mg/L) × 2.5.

**Table 2:** Method of intake.

Assigned Group	First Period (0–8 Weeks)	Wash-Out Period (8–11 Weeks)	Second Period (12–20 Weeks)
A	BDSW intake		Placebo intake
B	Placebo intake		BDSW intake

**Table 3:** Baseline of effectiveness criteria.

	BDSW Group (*n* = 37)	Placebo Group (*n* = 37)	*p*-Value ^1)^
FPG (mg/dL)	99.59 ± 7.96	99.14 ± 10.05	0.828
PPG_0.5h_ (mg/dL)	174.14 ± 18.68	177.65 ± 28.05	0.528
PPG_1.0h_ (mg/dL)	189.62 ± 28.57	192.00 ± 37.05	0.758
PPG_1.5h_ (mg/dL)	167.97 ± 37.67	176.30 ± 41.33	0.368
PPG_2h_ (mg/dL)	149.32 ± 33.18	146.92 ± 38.95	0.776
iAUC_0−2h_ (hr*mg/dL)	129.12 ± 37.88	136.47 ± 49.76	0.477
FPI (μU/mL)	8.66 ± 4.87	7.95 ± 3.88	0.494
HOMA-IR	2.13 ± 1.19	1.96 ± 1.03	0.510
C-peptide (ng/mL)	1.94 ± 0.70	1.91 ± 0.55	0.844
HbA1c (%)	5.82 ± 0.30	5.80 ± 0.32	0.766
TC (mg/dL)	204.30 ± 33.94	194.62 ± 34.08	0.225
TG (mg/dL)	138.81 ± 64.57	161.46 ± 100.70	0.254
HDL-C (mg/dL)	48.05 ± 8.93	47.22 ± 9.23	0.693
LDL-C (mg/dL)	128.41 ± 29.80	116.22 ± 33.00	0.100
Weight (kg)	66.37 ± 13.57	66.31 ± 13.71	0.986
BMI (kg/m^2^)	25.19 ± 4.01	25.16 ± 4.05	0.977
BFM (g)	20.01 ± 8.12	19.69 ± 8.31	0.868
PBF (%)	29.98 ± 7.20	29.58 ± 7.58	0.819
WC (cm)	88.67 ± 9.85	88.08 ± 9.82	0.797
HC (cm)	95.53 ± 9.17	95.36 ± 9.23	0.936
WHR	0.93 ± 0.05	0.92 ± 0.04	0.669

Values are presented as mean ± SD. FPG, fasting plasma glucose; PPG, postprandial plasma glucose; iAUC, glucose area; FPI, fasting plasma insulin; HOMA-IR, homeostatic model assessment of insulin resistance; HbA1c, glycosylated hemoglobin, type A1c; TC, total cholesterol; TG, triglycerides; HDL-C, high-density lipoprotein–cholesterol; LDL-C, low-density lipoprotein–cholesterol; BMI, body mass index; BFM, body fat mass; PBF, percent body fat; WC, waist measurement; HC, hip measurement; WHR, waist–hip ratio. ^1)^ Analyzed by independent t-test.

**Table 4:** Fasting and postprandial glucose changes before and after eight weeks of ingestion.

		BDSW Group (*n* = 37)				Placebo Group (*n* = 37)				*p*-Value ^2)^
		Baseline	8 Weeks	Change Value	*p*-Value ^1)^	Baseline	8 Weeks	Change Value	*p*-Value ^1)^	
FPG (mg/dL)		99.59 ± 7.96	100.32 ± 8.69	0.73 ± 4.88	0.369	99.14 ± 10.05	99.59 ± 7.79	0.46 ± 6.91	0.689	0.837
PPG (mg/dL)	30 min	174.14 ± 18.68	171.19 ± 20.69	−2.95 ± 22.28	0.427	177.65 ± 28.05	176.54 ± 20.99	−1.11 ± 26.92	0.804	0.734
	60 min	189.62 ± 28.57	183.65 ± 34.09	−5.97 ± 32.36	0.269	192.00 ± 37.05	186.30 ± 35.07	−5.70 ± 31.51	0.278	0.966
	90 min	167.97 ± 37.67	160.84 ± 37.62	−7.14 ± 35.34	0.227	176.30 ± 41.33	165.11 ± 34.25	−11.19 ± 38.46	0.085	0.657
	120 min	149.32 ± 33.18	141.86 ± 36.27	−7.46 ± 34.58	0.198	146.92 ± 38.95	136.03 ± 29.18	−10.89 ± 39.28	0.100	0.679
iAUC_0−2h_ (h·mg/dL)		129.12 ± 37.88	118.32 ± 39.24	−10.81 ± 35.23	0.070	136.47 ± 49.76	124.18 ± 40.45	−12.29 ± 43.32	0.093	0.900

Values are presented as mean ± SD. Change value = value at 8 weeks − baseline value. ^1)^ Analyzed by paired t-test compared within the group. ^2)^ Analyzed by independent t-test change values of the comparison between groups (analyzed by a linear mixed effect model for repeated measures data).

**Table 5:** Changes in blood glucose-related indicators before and after ingestion.

	BDSW Group (*n* = 37)				Placebo Group (*n* = 37)				*p*-Value ^2)^
	Baseline	8 Weeks	Change Value	*p*-Value ^1)^	Baseline	8 Weeks	Change Value	*p*-Value ^1)^	
FPI (μU/mL)	8.66 ± 4.87	7.59 ± 3.87	−1.07 ± 3.97	0.110	7.95 ± 3.88	8.62 ± 4.14	0.67 ± 2.76	0.151	0.042 *
HOMA-IR	2.13 ± 1.19	1.87 ± 0.92	−0.27 ± 1.01	0.113	1.96 ± 1.03	2.13 ± 1.03	0.17 ± 0.70	0.157	0.049 *
C-peptide (ng/mL)	1.94 ± 0.70	1.87 ± 0.49	−0.07 ± 0.53	0.452	1.91 ± 0.55	1.96 ± 0.57	0.05 ± 0.30	0.311	0.263
HbA1c (%)	5.82 ± 0.30	5.83 ± 0.32	0.01 ± 0.17	0.846	5.80 ± 0.32	5.84 ± 0.31	0.04 ± 0.15	0.088	0.249

Values are presented as mean ± SD. * *p* <0.05. Change value = value at 8 weeks − baseline value. ^1)^ Analyzed by paired t-test compared within the group. ^2)^ Analyzed by independent t-test change values of the comparison between groups (analyzed by a linear mixed effect model for repeated measures data).

**Table 6:** Changes in blood lipid profile before and after ingestion.

	BDSW group (*n* = 37)				Placebo group (*n* = 37)				*p*-Value ^2)^
	Baseline	8 Weeks	Change Value	*p*-Value ^1)^	Baseline	8 Weeks	Change Value	*p*-Value ^1)^	
TC (mg/dL)	204.30 ± 33.94	198.62 ± 33.21	−5.68 ± 28.70	0.237	194.62 ± 34.08	205.03 ± 33.71	10.41 ± 23.02	0.009 **	0.006 **
TG (mg/dL)	138.81 ± 64.57	141.68 ± 81.61	2.86 ± 78.97	0.827	161.46 ± 100.70	140.14 ± 78.78	−21.32 ± 78.09	0.105	0.184
HDL-C (mg/dL)	48.05 ± 8.93	49.14 ± 9.48	1.08 ± 7.34	0.376	47.22 ± 9.23	49.95 ± 9.36	2.73 ± 5.35	0.004 **	0.289
LDL-C (mg/dL)	128.41 ± 29.80	121.19 ± 32.86	−7.22 ± 28.44	0.131	116.22 ± 33.00	127.22 ± 29.21	11.00 ± 23.68	0.008 **	0.003 **

Values are presented as mean ± SD. ** *p* < 0.01. Change value = value at 8 weeks − baseline value. ^1)^ Analyzed by paired t-test compared within the group. ^2)^ Analyzed by an independent t-test change values of the comparison between groups (analyzed by a linear mixed effect model for repeated measures data).

**Table 7:** Dietary intake analysis before and after ingestion.

	BDSW group (*n* = 37)				Placebo group (*n* = 37)				*p*-Value ^2)^
	Baseline	8 Weeks	Change Value	*p*-Value ^1)^	Baseline	8 Weeks	Change Value	*p*-Value ^1)^	
Calorie (kcal)	1676.28 ± 410.77	1721.93 ± 449.86	19.35 ± 384.25	0.647	1767.29 ± 563.25	1753.15 ± 454.26	−14.14 ± 495.96	0.863	0.622
Carbohydrate (g)	251.27 ± 68.93	263.63 ± 78.52	7.82 ± 60.88	0.367	259.77 ± 82.75	256.24 ± 71.15	−3.53 ± 72.32	0.768	0.344
Fat (g)	42.82 ± 16.22	41.13 ± 16.83	−2.47 ± 17.75	0.502	44.52 ± 22.18	45.12 ± 17.60	0.60 ± 24.77	0.884	0.624
Protein (g)	68.72 ± 19.28	69.32 ± 22.72	0.01 ± 22.70	0.927	71.02 ± 23.91	72.40 ± 20.32	1.38 ± 24.71	0.735	0.910
Dietary fiber (g)	22.83 ± 8.19	24.23 ± 8.48	1.24 ± 7.30	0.290	24.67 ± 9.24	24.39 ± 8.88	−0.28 ± 8.40	0.843	0.265

Values are presented as mean ± SD. Change value = value at 8 weeks − baseline value. ^1)^ Analyzed by paired t-test compared within the group. ^2)^ Analyzed by independent t-test change values of the comparison between groups (analyzed by a linear mixed effect model for repeated measures data).

**Table 8:** Laboratory medicinal examination.

	BDSW Group (*n* = 40)				Placebo Group (*n* = 40)				*p*-Value ^2)^
	Baseline	8 Weeks	Change Value	*p*-Value ^1)^	Baseline	8 Weeks	Change Value	*p*-Value ^1)^	
**Hematology**									
WBC (×10^3^/μL)	5.90 ± 1.74	5.76 ± 1.60	−0.14 ± 1.41	0.547	5.77 ± 1.52	5.70 ± 1.41	−0.07 ± 0.84	0.598	0.807
RBC (×100^3^/μL)	4.51 ± 0.34	4.52 ± 0.33	0.01 ± 0.15	0.831	4.51 ± 0.33	4.53 ± 0.35	0.02 ± 0.15	0.392	0.655
Hemoglobin (g/dL)	13.80 ± 1.11	13.84 ± 1.05	0.04 ± 0.51	0.667	13.85 ± 1.10	13.94 ± 1.14	0.09 ± 0.51	0.296	0.654
Hematocrit (%)	40.91 ± 2.75	41.08 ± 2.79	0.17 ± 1.38	0.440	40.97 ± 2.99	41.15 ± 3.03	0.18 ± 1.37	0.418	0.982
Platelet (×10^3^/μL)	276.85 ± 72.02	277.98 ± 62.85	1.13 ± 24.97	0.777	272.73 ± 66.17	279.40 ± 60.35	6.68 ± 25.20	0.102	0.351
**Biochemistry**									
ALP (IU/L)	68.85 ± 18.24	68.18 ± 16.96	−0.68 ± 6.66	0.525	68.13 ± 17.80	68.00 ± 19.18	−0.13 ± 7.38	0.915	0.736
GGT (IU/L)	28.93 ± 18.73	27.68 ± 18.52	−1.25 ± 8.21	0.342	28.53 ± 18.92	27.33 ± 19.18	−1.20 ± 8.78	0.393	0.979
AST (IU/L)	24.15 ± 5.78	24.78 ± 6.29	0.63 ± 4.35	0.369	23.78 ± 5.70	23.43 ± 6.08	−0.35 ± 5.88	0.709	0.427
ALT (IU/L)	25.95 ± 9.06	27.10 ± 10.99	1.15 ± 7.99	0.368	25.40 ± 8.94	24.53 ± 7.49	−0.88 ± 6.55	0.404	0.262
Total bilirubin (mg/dL)	0.91 ± 0.35	0.86 ± 0.30	−0.05 ± 0.24	0.189	0.86 ± 0.32	0.88 ± 0.27	0.01 ± 0.23	0.707	0.209
Total protein (g/dL)	7.32 ± 0.36	7.28 ± 0.31	−0.04 ± 0.27	0.359	7.28 ± 0.36	7.34 ± 0.33	0.07 ± 0.30	0.176	0.101
Albumin (g/dL)	4.26 ± 0.19	4.25 ± 0.20	−0.01 ± 0.16	0.841	4.22 ± 0.17	4.27 ± 0.18	0.04 ± 0.14	0.068	0.179
BUN (mg/dL)	15.28 ± 3.69	15.20 ± 3.63	−0.08 ± 3.72	0.899	15.60 ± 3.48	14.60 ± 4.15	−1.00 ± 4.20	0.139	0.308
Creatinine (mg/dL)	0.62 ± 0.14	0.63 ± 0.13	0.01 ± 0.13	0.698	0.63 ± 0.14	0.64 ± 0.14	0.01 ± 0.07	0.642	0.910
eGFR (mL/min/1.7)	107.93 ± 8.22	106.58 ± 8.30	−1.35 ± 11.49	0.462	106.62 ± 8.03	106.29 ± 8.18	−0.33 ± 5.05	0.686	0.582
**Urinary**									
SG	1.02 ± 0.00	1.02 ± 0.01	0.00 ± 0.01	0.570	1.02 ± 0.00	1.02 ± 0.00	0.00 ± 0.01	0.517	0.985
pH	6.14 ± 0.72	5.89 ± 0.72	−0.25 ± 0.74	0.040 *	6.30 ± 0.86	6.09 ± 0.75	−0.21 ± 0.78	0.094	0.817

Values are presented as mean ± SD. * *p* < 0.05. Change value = value at 8 weeks − baseline value. WBC, white blood cell; RBC, red blood cell; ALP, alkaline phosphatase; GGT, gamma-glutamyl transferase; AST, aspartate aminotransferase; ALT, alanine aminotransferase; BUN, blood urea nitrogen; eGFR, estimated glomerular filtration rate; SG, specific gravity. ^1)^ Analyzed by paired t-test compared within the group. ^2)^ Analyzed by linear mixed effect model for repeated measures data.

**Table 9:** Vital signs.

	BDSW Group (*n* = 40)				Placebo Group (*n* = 40)				*p*-Value ^2)^
	Baseline	8 Weeks	Change Value	*p*-Value ^1)^	Baseline	8 Weeks	Change Value	*p*-Value ^1)^	
SBP (mmHg)	119.65 ± 12.46	120.83 ± 10.70	1.18 ± 11.40	0.518	120.03 ± 11.89	121.70 ± 10.43	1.68 ± 9.59	0.277	0.827
DBP (mmHg)	77.90 ± 8.95	78.98 ± 9.27	1.08 ± 6.66	0.314	77.38 ± 9.83	79.95 ± 7.56	2.58 ± 5.70	0.007 **	0.324
Pulse (number/min)	71.80 ± 9.91	72.20 ± 10.37	0.40 ± 8.92	0.778	71.70 ± 9.49	73.10 ± 9.42	1.40 ± 5.55	0.118	0.556

Values are presented as mean ± SD. ** *p* < 0.01. Change value = value at 8 weeks – baseline value. SBP, systolic blood pressure; DBP, diastolic blood pressure. ^1)^ Analyzed by paired t-test compared within group. ^2)^ Analyzed by linear mixed effect model for repeated measures data.

## Supplementary Materials

The following are available online at https://www.mdpi.com/2072-6643/12/2/515/s1, Supplementary File 1. Criterion for exclusion of study subjects; Supplementary File 2. Schedule summary; Supplementary File 3. Baseline demographic and clinical information.

## Appendix A

**Table A1:** CONSORT 2010 checklist of information to include when reporting a randomized trial.

Section/Topic	Item No	Checklist Item	Reported on Page No.
**Title and abstract**			
	1a	Identification as a randomized trial in the title	1
	1b	Structured summary of trial design, methods, results, and conclusions (for specific guidance see CONSORT for abstracts)	1
**Introduction**			
Background and objectives	2a	Scientific background and explanation of the rationale	1, 2
	2b	Specific objectives or hypotheses	2
**Methods**			
Trial design	3a	Description of trial design (such as parallel, factorial) including allocation ratio	4, Figure A1
	3b	Important changes to methods after trial commencement (such as eligibility criteria), with reasons	*n*/A
Participants	4a	Eligibility criteria for participants	2, 3, Suppl. File 1
	4b	Settings and locations where the data were collected	2
Interventions	5	The interventions for each group with sufficient details to allow replication, including how and when they were actually administered	2, 4, 5, Suppl. File 2
Outcomes	6a	Completely defined pre-specified primary and secondary outcome measures, including how and when they were assessed	4, 5, Suppl. File 2
	6b	Any changes to trial outcomes after the trial commenced, with reasons	*n*/A
Sample size	7a	How sample size was determined	5, Figure A1
	7b	When applicable, explanation of any interim analyses and stopping guidelines	*n*/A
Randomization:			
Sequence generation	8a	Method used to generate the random allocation sequence	4, Figure A1
	8b	Type of randomization; details of any restriction (such as blocking and block size)	4, Figure A1
Allocation concealment mechanism	9	Mechanism used to implement the random allocation sequence (such as sequentially numbered containers), describing any steps taken to conceal the sequence until interventions were assigned	4
Implementation	10	Who generated the random allocation sequence, who enrolled participants, and who assigned participants to interventions	4
Blinding	11a	If done, who was blinded after assignment to interventions (for example, participants, care providers, those assessing outcomes) and how	4
	11b	If relevant, description of the similarity of interventions	*n*/A
Statistical methods	12a	Statistical methods used to compare groups for primary and secondary outcomes	5
	12b	Methods for additional analyses, such as subgroup analyses and adjusted analyses	5
**Results**			
Participant flow (a diagram is strongly recommended)	13a	For each group, the numbers of participants who were randomly assigned, received intended treatment, and were analyzed for the primary outcome	5, Figure A1
	13b	For each group, losses and exclusions after randomization, together with reasons	5, Figure A1
Recruitment	14a	Dates defining the periods of recruitment and follow-up	Suppl. File 2
	14b	Why the trial ended or was stopped	*n*/A
Baseline data	15	A table showing baseline demographic and clinical characteristics for each group	Suppl. File 3, Table 3
Numbers analyzed	16	For each group, number of participants (denominator) included in each analysis and whether the analysis was by original assigned groups	5, Figure A1
Outcomes and estimation	17a	For each primary and secondary outcome, results for each group, and the estimated effect size and its precision (such as 95% confidence interval)	5–8, Table 4, Table 5, and Table 6
	17b	For binary outcomes, presentation of both absolute and relative effect sizes is recommended	*n*/A
Ancillary analyses	18	Results of any other analyses performed, including subgroup analyses and adjusted analyses, distinguishing pre-specified from exploratory	*n*/A
Harms	19	All important harms or unintended effects in each group (for specific guidance see CONSORT for harms)	*n*/A
**Discussion**			
Limitations	20	Trial limitations, addressing sources of potential bias, imprecision, and, if relevant, multiplicity of analyses	*n*/A
Generalizability	21	Generalizability (external validity, applicability) of the trial findings	11, 12
Interpretation	22	Interpretation consistent with results, balancing benefits and harms, and considering other relevant evidence	11, 12
Other information			
Registration	23	Registration number and name of trial registry	4
Protocol	24	Where the full trial protocol can be accessed, if available	*n*/A
Funding	25	Sources of funding and other support (such as supply of drugs), role of funders	12
