# Supplementary material for: Natural Magnesium-Enriched Deep-Sea Water Improves Insulin Resistance and the Lipid Profile of Prediabetic Adults: A Randomized, Double-Blinded Crossover Trial
Source: Nutrients. 2020 Feb 18;12(2):515. doi: 10.3390/nu12020515 (PMC7071302; doi:10.3390/nu12020515)
Supplement: Supplementary file 1 [file nutrients-12-00515-s001.pdf]

Supplementary Materials

Supplementary File 1. Criterion for exclusion of subjects

Subjects were excluded from participating in the clinical trial if any of the following criteria were met; (1) those who lost more than 10% of their weight within the last 3 months, (2) those diagnosed with type 1 or type 2 diabetes, (3) those with poor blood glucose control by the HbA1c 9.0% or more, (4) those with systolic blood pressure of more than 160 mmHg or diastolic blood pressure of more than 100 mmHg (however, those who were able to control blood pressure with medication were allowed to participate), (5) those taking a whole body corticosteroid within 4 weeks of the first dose of the trial product, (6) those who had taken medications such as hypoglycemic agents, obesity drugs, or lipid-lowering drugs within the last 6 months or medicines and health functional foods that could affect the interpretation of the results of this study, including health functional foods related to blood sugar, obesity, and lipid improvement within 2 months, (7) those with heart failure, myocardial infarction, stroke, or other acute and severe cardiovascular disease, (8) those with local or systemic inflammatory diseases such as rheumatoid arthritis or other autoimmune diseases, (9) those with hereditary hyperlipidemia, renal disease such as acute/chronic renal failure, nephrotic syndrome, cancer, autoimmune disease, respiratory disease, (10) those with alcoholism, drug abuse or dependence, gastrointestinal disorders (eg, Crohn's disease) or those having received gastrointestinal surgery (excluding simple appendectomy or hernia surgery) that may affect the absorption of trial products, (11) those who had a history of hypersensitivity or clinically significant hypersensitivity reactions to drugs and human application products, (12) those who participated in another trial within 2 months of the screening trial, pregnant or lactating women, (13) those whose medical examination shows the following results: AST or ALT more than twice the upper reference limit, or serum creatine of 2.0 mg/dL or higher.

Supplementary File 2. Schedule summary

Schedule		Screening	First period		Wash-out period	Second period	
Items		Within -3 weeks	First visit<br>0 week	Second visit<br>8 weeks	8-11 weeks	Third visit<br>12 weeks	Forth visit<br>20 weeks
Written consent		●					
Demographic information · the history of a disease survey		●					
Drug-possession survey		●	●	●		●	●
Medical condition change survey			●				
Drinking · smoking survey		●		●		●	●
Selection and exclusion criteria check		●	●				
Random assignment			●				
Physical examination, medicinal examination, HCG		●		●		●	●
Electrocardiogram		●					●
Vital signs		●	●	●		●	●
Blood glucose -related index	75 g OGTT (FPG, PPG, iAUC)	●	●	●		●	●
	HbA1c	●		●		●	●
	Fasting insulin, HOMA-IR, C-peptide		●	●		●	●
Blood lipid index	TC, TG, HDL-C, LDL-C	●		●		●	●
Physical measures	Height, weight	●					
	Weight, body fat, body fat percentage, body mass index, WC, HC, WHR		●	●		●	●
Sample banking for examination of human derivatives (epinephrine, glucocorticoid, adiponectin, omentin)			●	●		●	●
Provision of products for clinical trial			●			●	
Dietary record distribution		●	●	●		●	
Survey on eating and physical activity			●	●		●	●
Collection of returned products, compliance evaluation				●			●
Adverse reaction survey				●		●	●

HCG, human chorionic gonadotropin; OGTT, oral glucose tolerance test; FPG, Fasting plasma glucose; PPG, postprandial plasma glucose; iAUC, glucose area; HbA1c, glycosylated hemoglobin, type A1c; HOMA-IR, homeostatic model assessment of insulin resistance; TC, total cholesterol; TG, triglycerides; HDL-C, high density lipoprotein–cholesterol; LDL-C, low density lipoprotein-cholesterol; WC, waist measurement; HC, hip measurement; WHR, waist-hip ratio

Supplementary File 3. Baseline demographic and clinical information

Baseline item		Total (n=40)	
Sex (n)	Male	17 (42.5%)	
	Female	23 (57.5%)	
Age (years)		52.88±6.53	
Height (cm)		162.18±8.45	
Weight (kg)		67.76±14.22	
BMI (kg/m^2)		25.63±4.27	
BFM (g)		20.15±8.30	
PBF (%)		29.94±7.33	
WC (cm)	Male (n=17)	91.56±8.81	88.75±10.17
	Female (n=23)	86.68±10.78	
HC (cm)	Male (n=17)	97.55±5.89	95.78±9.08
	Female (n=23)	94.47±10.80	
WHR		0.93±0.05	
SBP (mmHg)		121.03±13.01	
DBP (mmHg)		78.50±9.13	
Pulse (number/min)		70.25±8.76	
Drinker No. (%)	Yes	19 (47.5%)	
	No	21 (52.5%)	
Alcohol consumption (unit/weeks)		7.27±8.10	
Smoker No. (%)	Yes	4 (10%)	
	No	36 (90%)	
Amount of smoking (cigarette/day)		12.75±4.86	
Glucose	FPG (mg/dL)	97.63±8.85	
	2h-PPG (mg/dL)	159.48±26.65	
HbA1c (%)		5.83±0.32	

Values are presented as mean ± SD

BMI, body mass index; BFM, body fat mass; PBF, percent body fat; WC, waist measurement; HC, hip measurement; WHR, waist-hip ratio; SBP, systolic blood pressure; DBP, diastolic blood pressure; FPG, Fasting plasma glucose; 2h-PPG, 2 hour postprandial plasma glucose; HbA1c, glycosylated hemoglobin, type A1c.
